# Predicting outcomes in selective fetal growth restriction of monoChOrioNic Twins: an inteRnAtional observational cohort STudy protocol (CONTRAST study)

**DOI:** 10.1136/bmjopen-2025-114000

**Published:** 2026-02-24

**Authors:** Anne Noll, Ali Javinani, Femke Slaghekke, Monique C Haak, Jeanine van Klink, Lotte Van der Meeren, Enrico Lopriore, Francesca Russo, Michael Aertsen, Alireza Shamshirsaz, S Shinar, Mar Bennasar, Eleonor Tiblad, Lotta Herling, Liesbeth Lewi, EJT (Joanne) Verweij, CONTRAST Study Group, Laura de Keizer

**Affiliations:** 1 Department of Obstetrics and Gynecology, Division of Fetal Medicine, Leiden Universitair Medisch Centrum, Leiden, The Netherlands; 2 Department of Obstetrics and Gynecology, University Hospitals Leuven, Leuven, Belgium; 3 Center for Fetal Medicine, Karolinska University Hospital, Stockholm, Sweden; 4 Maternal Fetal Care Center, Boston Children’s Hospital, Harvard Medical School, Boston, Massachusetts, USA; 5 Neonatology, Willem-Alexander Children's Hospital, Department of Pediatrics, Leiden University Medical Center, Leiden, Netherlands; 6 Department of Pathology, Leiden Universitair Medisch Centrum, Leiden, The Netherlands; 7 Department of Pathology, Erasmus MC Universitair Medisch Centrum Rotterdam, Rotterdam, The Netherlands; 8 Department of Development and Regeneration, Biomedical Sciences, KU leuven, Leuven, Belgium; 9 Department of Imaging and Pathology, University Hospitals Leuven, Leuven, Belgium; 10 Ontario Fetal Center, Division of Maternal Fetal Medicine, Department of Obstetrics and Gynaecology, Mount Sinai Hospital, University of Toronto, Toronto, Ontario, Canada; 11 BCNatal, Hospital Clinic and Hospital Sant Joan de Deu, Barcelona, Spain; 12 Clinical Epidemiology Division, Karolinska Institute, Stockholm, Sweden; 13 Center for Fetal Medicine, Pregnancy Care and Delivery, Karolinska University Hospital, Stockholm, Sweden; 14 Department of Clinical Science, Intervention and Technology, Karolinksa Institutet, Stockholm, Sweden

**Keywords:** Fetal medicine, Clinical Protocols, OBSTETRICS, Observational Study, ULTRASONOGRAPHY

## Abstract

**Introduction:**

Selective fetal growth restriction (sFGR) is a major cause of perinatal morbidity and mortality in monochorionic diamniotic (MCDA) twin pregnancies. Current management relies on umbilical artery Doppler patterns in the smaller twin. These patterns are, however, inconsistent and do not represent a reliable severity scale, complicating clinical decision-making and parental counselling. This study aims to improve risk stratification by identifying predictors of adverse outcomes, while also evaluating the pathophysiology and multi-organ impact of sFGR in early childhood.

**Methods and analysis:**

This is a prospective, international, multicentre cohort study conducted in six tertiary fetal medicine centres with expertise in complicated twin pregnancies. Recruitment began in March 2023 and will continue until December 2026, targeting 274 MCDA twin pairs with complete follow-up to develop a prediction model for adverse perinatal outcomes in sFGR at the time of diagnosis. Standardised data collection includes serial ultrasound examinations, advanced fetal imaging (cardiac, cerebral and 3D volumetric), fetal brain MRI and detailed placental phenotyping. Maternal and parental well-being are assessed during pregnancy and after birth. Neurodevelopmental outcome is evaluated up to 2 years after birth using validated tools. The statistical analysis plan includes predictive modelling with internal validation.

**Ethics and dissemination:**

The study has been approved by the ethical review boards of all participating centres. Findings will be disseminated through peer-reviewed publications, international conferences and engagement with clinical guideline committees.

**Trial registration number:**

NCT05952583.

**STRENGTHS AND LIMITATIONS OF THIS STUDY**

- Prospective, international multicentre cohort design with standardised study data collection across six fetal medicine centres.
- Harmonised, quality-controlled imaging protocol across centres, with regular audits, including advanced ultrasound, 3D volumetry and fetal MRI.
- Standardised placental phenotyping and parental well-being assessments from prenatal to postnatal stages.
- Observational design, limiting causal inference.
- Recruitment in tertiary care centres may be biased towards severe cases; international differences in referral/management and loss to follow-up are anticipated.

## Introduction

 Selective fetal growth restriction (sFGR) complicates approximately 15%–20% of monochorionic diamniotic (MCDA) twin pregnancies and is defined by substantial discordant growth between the twins.^1^ It is associated with high risks of intrauterine death and severe neonatal morbidity in one or both twins.^1^ Management is particularly challenging, as clinicians must weigh the benefits of prolonging gestation against the risks of fetal deterioration, death and morbidity in the co-twin resulting from a very preterm delivery. Although fetal interventions such as selective reduction or cord occlusion may be considered in selected severe cases, no curative treatment exists. As no curative treatment exists, timely delivery to save both twins remains the only intervention, making accurate outcome prediction essential.

Current management is based on the classification of umbilical artery Doppler patterns in the smaller twin (type I to III).^2^ Although associated with distinct prognoses, these patterns can vary considerably during pregnancy and do not represent a progressive severity scale.^2^,^12^ This uncertainty complicates both delivery timing and parental counselling. Improved risk stratification is therefore needed. A severity-based prediction model that integrates ultrasound parameters could better distinguish between mild and severe clinical courses, supporting individualised management, more precise counselling and ultimately improved outcomes.

In addition to improving prediction of the clinical course, it is also important to recognise that the effects of sFGR extend beyond growth itself, with potential consequences for vital organ development and long-term outcomes. Limited evidence suggests cardiac adaptation in both twins, including hypertrophy in the larger twin and impaired ventricular function in the smaller twin, as well as an increased risk of congenital heart disease.^13^,^15^ Neurodevelopmental effects are even less well understood. In singletons with growth restriction, altered brain structure and connectivity have been demonstrated, but systematic evaluation in MCDA twin pregnancies is lacking.^16 17^ Emerging data suggest impaired neurodevelopment in growth-restricted twins,^18^ yet outcomes in single survivors remain largely unknown. The genetically identical co-twin design offers a unique opportunity to study these effects while controlling for genetic and gestational confounders.

Another important but incompletely understood aspect of sFGR is its pathophysiology. Unequal placental sharing plays a central role, leaving one twin with reduced nutrient and oxygen supply.^19^,^22^ Yet, sFGR develops only in a proportion of such pregnancies,^23^ suggesting that additional mechanisms are involved. These may include differences in placental vascular architecture: type III placentas often feature large artery-to-artery anastomoses and extensive shared intertwin circulation, whereas these are typically absent in type II.^2024^,^26^ Whether placental dysfunction—particularly affecting the smaller twin—contributes to the pathophysiology of sFGR remains unclear. However, systematic histopathological studies have not yet been conducted, and correlating these with antenatal imaging could provide valuable insights into disease mechanisms.

Finally, the psychological impact of sFGR on parents remains largely unexplored. Twin pregnancies are already associated with increased stress and depressive symptoms,^27 28^ yet little is known about how sFGR affects parental well-being and attachment patterns before and after birth.

To address these gaps, the CONTRAST study brings together leading fetal medicine centres in Europe and North America. Its primary aim is to develop a prediction model for adverse outcomes in sFGR at the time of diagnosis. Secondary aims are to evaluate the impact on fetal organ development and long-term neurodevelopment, investigate associations between antenatal findings, placental pathology and outcome, and examine parental psychological well-being and attachment. This prospective collaboration seeks to advance the understanding, classification and management of sFGR and ultimately improve outcomes for affected families.

## Methods and analysis

### Study design and setting

The CONTRAST study is an ongoing international, prospective, multicentre cohort study in MCDA twin pregnancies complicated by sFGR. Six tertiary fetal medicine centres participate: Leiden University Medical Center (Netherlands), University Hospitals Leuven (Belgium), Karolinska University Hospital (Sweden), BCNatal (Spain), Boston Children’s Hospital (USA) and Mount Sinai/Hospital for Sick Children (Canada).

### Eligibility

We include MCDA twin pregnancies with sFGR diagnosed before 28 weeks’ gestation, regardless of umbilical artery Doppler flow pattern. sFGR is defined as a persistent (observed during ≥two consecutive ultrasound examinations) estimated fetal weight (EFW) discordance of ≥20% between the twins, in the absence of twin-to-twin transfusion syndrome (TTTS) or twin anaemia–polycythaemia sequence (TAPS) at time of inclusion.

This sFGR definition enables consistent inclusion across centres regardless of growth chart usage and aligns with clinical practice, whereas the more complex Delphi consensus criteria^29^ have shown limited adoption and similar predictive accuracy.^30^ Recent data further support intertwin EFW discordance as a stronger predictor of adverse outcome than centile-based definitions in monochorionic twins.^31^ An EFW discordance threshold of ≥20% is associated with increased perinatal risk and robust predictive performance,^31^,^34^ allowing consistent application across centres. The upper inclusion limit of 28 weeks’ gestation was selected to capture both early and later presentations of sFGR, thereby enhancing the clinical relevance and generalisability of the prediction model.

- EFW discordance is calculated as (EFW larger twin–EFW smaller twin)/EFW larger twin×100.
- TTTS is defined by the presence of discordant amniotic fluid volumes, with a maximum vertical pocket >6 cm before 16 weeks, >8 cm at 16–20 weeks or >10 cm after 20 weeks in one sac and <2 cm in the co-twin, accompanied by a distended bladder (defined as persistently visible and subjectively enlarged on ultrasound) in the recipient and an absent or small bladder in the donor twin.^35^,^37^
- TAPS is defined by a discordance in middle cerebral artery peak systolic velocity >0.5 multiples of the median, which may be accompanied by supportive features such as placental dichotomy or a ‘starry-sky’ appearance of the fetal liver in the anaemic twin.^38^

Both parents must be ≥18 years and able to provide informed consent. Pregnancies with fetuses affected by lethal anomalies or explicitly referred for a fetal intervention without follow-up are excluded.

### Study procedures

**Figure 1:**
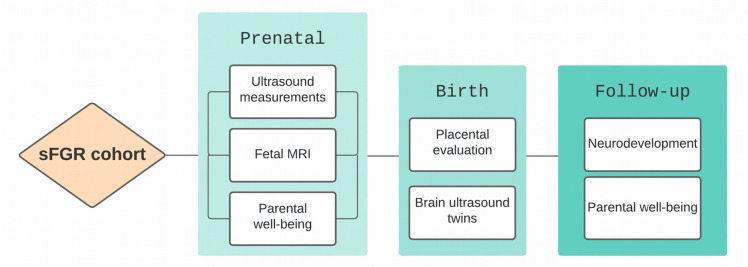
An overview of the main assessments that participants in the sFGR cohort will undergo during the research. sFGR, selective fetal growth restriction.

Participants are followed from diagnosis at the tertiary care centre until 24 months of age, corrected for premature birth, with data collected across three phases (figure 1). Throughout the study, collaboration within a multidisciplinary team—including fetal medicine specialists, perinatal pathologists, neonatologists and paediatric neuropsychologists—supports consistent data collection and strengthens both study design and interpretation.

### Phase I: prenatal

**Figure 2:**
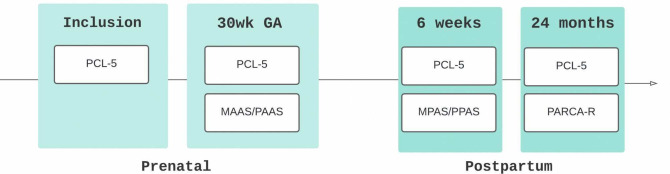
Overview of psychological assessments. GA, gestational age; MAAS, Maternal Antenatal Attachment Scale; MPAS, Maternal Postnatal Attachment Scale; PAAS, Paternal Antenatal Attachment Scale; PARCA-R, Parent Report of Children’s Abilities-Revised; PCL-5, Post-Traumatic Stress Syndrome Checklist for DSM-5; PPAS, Paternal Postnatal Attachment Scale.

All participants receive routine clinical care for MCDA twins according to local protocols. In addition, standardised ultrasound measurements such as umbilical venous Doppler flow, cardiac function, assessment of placental structure and fetal brain development are collected at inclusion (T1), 20–22 weeks (T2) and 28–30 weeks’ gestation (T3) (table 1). If inclusion coincides with the second time point (20–22 weeks), only two assessments are performed. A full list of the ultrasound parameters is provided in table 2. Fetal MRI is scheduled at 28–30 weeks’ gestation, currently implemented at selected centres, with ongoing expansion planned across all sites. The acquisition details of the different antenatal images are detailed in table 2. Regular quality audits are performed to monitor imaging, data entry and adherence to study procedures, thereby ensuring consistency and data integrity. Parental psychological well-being is assessed using validated questionnaires at inclusion and at 30 weeks of gestation (figure 2).

**Table 1:** Timing of ultrasound and fetal MRI assessments

GA (weeks)	Standard measurements*	Additional measurements				
		Flow	Placental assessment	Cardiac assessment	Cerebral assessment	Fetal MRI
Inclusion (<28) (T1)	Routine ultrasound (local protocol)	UV: flow and diameter	AA-anastomosis: flow and diameter Placental appearance	CT-ratio In/outflow times TV regurgitation 4C-clip		
20–22 (T2)	Routine ultrasounds (local protocol)	UV: flow and diameter	Placental appearance	CT-ratio In/outflow times TV regurgitation 4C-clip	Still images 3D volume (20–24 weeks)	
22–28	Routine ultrasounds (local protocol)					
28–30 (T3)	Routine ultrasounds (local protocol)	UV: flow and diameter	Placental appearance	CT-ratio In/outflow times TV regurgitation 4C-clip	Still images (28–32 weeks) 3D volume (28+0 weeks)	Fetal MRI (28–30 weeks)
30–delivery	Routine ultrasounds (local protocol)					

* Standard measurements are tailored to local protocol and patient needs.

4C, four-chamber view; CT, cardio-thoracic; 3D, three-dimensional; GA, gestational age; TV, tricuspid valve; UV, umbilical vein.

**Table 2:** Summary of image acquisition

Parameter	Acquisition details*		
Umbilical vein (UV)	Diameter: transverse abdominal section with spine left/right, UV horizontal; measure inner–inner vessel wall edges.	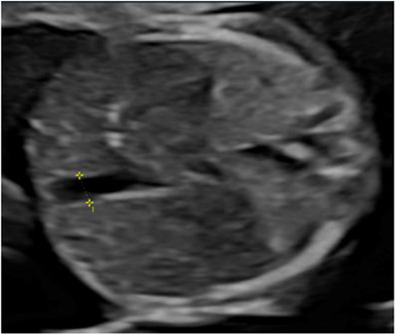	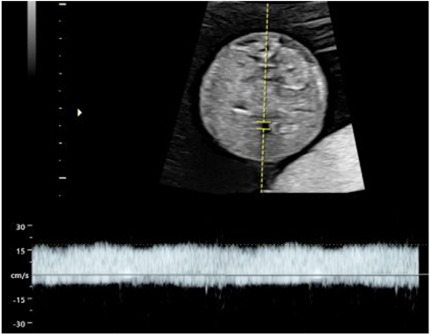
	Flow: spine up/down; pulsed-wave Doppler in mid-abdominal portion; insonation angle ≤30° (ideally 0°).		
		UV diameter (left) and UV flow right).	
Artery-to-artery (AA)	Placenta aligned with insonation angle; use color Doppler to detect bidirectional flow pattern; placenta perpendicular for diameter measurement.		
	Placental appearance	Sweep placenta; assess contrasting appearance (thickness and heterogeneity) between shares.		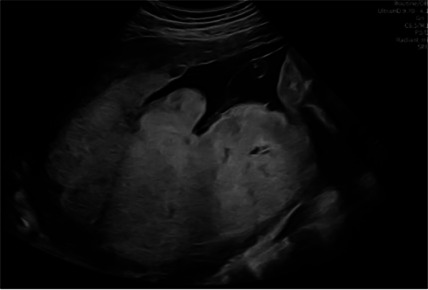
Cardiothoracic ratio (CT ratio)	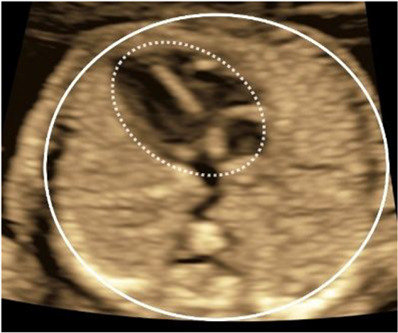	4C transverse view with whole thorax visible: horizontal septum, complete rib, no abdominal contents/skin. Measure at end-diastole (closed valves). CT ratio = heart circumference / thorax circumference.	
		Continuous ellipse: thoracic circumference Discontinuous ellipse: cardiac circumference	
Four chamber clip (4C clip)	4C apical/basal; clear myocardial walls; ≥60 frames/s; record ≥5–6 s clip with good angle and high frame rate. Clip of at least 5-6 seconds with good angle and high frame rate		
Inflow/ outflow times	Angle 0° if possible (no angle correction). Capture whole cardiac cycle (3–5 waves). Measure: velocity (E, A waves), inflow time, peak velocity (outflow), cycle time	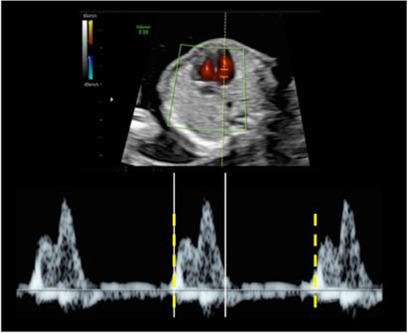	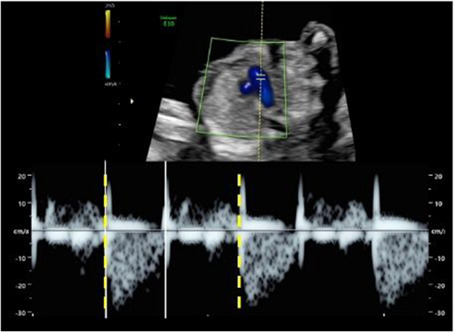
	White line: Inflow/Outflow time (respectively left and right image). Yellow line: whole cycle time.		
Tricuspid valve regurgitation	Pulsed-wave Doppler across TV, angle <30° to septum; high sweep speed; measure peak velocity (cm/s).		
Cerebral stills: transverse plane	Transthalamic plane: head circumference. Transventricular plane: both ventricles. Transcerebellar plane: transcerebellar diameter + cisterna magna.		
	Transthalamic plane for measurement of head circumference . Transcerebellar plane for measurement of transcerebellar diameter and cisterna magna. Transventricular plane for measurement of distal lateral ventricular width.		
	Cerebral stills: midsagittal plane	Measure: CC length (in–in), fastigium–CC genu, pons width (AP), vermian height (sup–inf lobule).		
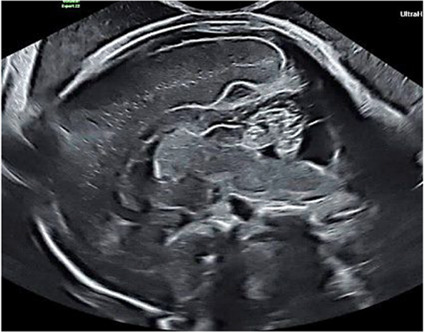		Midsagittal plane for measurement of corpus callosum length, corpus callosum to fastigium distance, vermian height and pons diameter.	
3D cerebral volume	Central axial at thalami; insonation angle ~70° to include skull. Acquire (~4 seconds) 3D volume per twin		
Fetal MRI core sequences	T2-weighted (axial/coronal/sagittal, 2–4 mm slice). T1-weighted (transverse). Diffusion-weighted (1–2 planes). Hemosiderin-sensitive (SWI or T2* EPI). 3D volumetric sequences.		

* Five centres use Voluson E22 machines (GE Healthcare) for ultrasound acquisition and one centre uses Canon APLIO i800/i700 machines (Canon Medical Systems). Fetal MRI scanner characteristics are recorded per examination. Standardised acquisition protocols and regular quality audits are used to ensure consistency across centres.

AP, anteroposterior; CC, corpus callosum; DWI, diffusion-weighted imaging; EPI, echo planar imaging; E wave, early ventricular filling wave; SSFSE, single-shot fast spin-echo; SWI, susceptibility-weighted imaging; T1, T1-weighted sequence; T2, T2-weighted sequence.

### Phase II: postnatal

After birth, delivery data are recorded, and fresh placentas undergo vascular dye injection followed by histological examination in accordance with the Amsterdam Consensus Criteria.^39^ Neonates receive cerebral ultrasound within 1–3 days to evaluate brain maturation and detect intracranial abnormalities.

### Phase III: follow-up

At 24 months’ corrected age, neurodevelopment is assessed using the Parent Report of Children’s Abilities-Revised (PARCA-R) questionnaire (primary assessment tool) for all children, and (if applicable) Bayley-III/IV testing and neurological examination by trained personnel, according to national neonatal follow-up guidelines.^40^ Severe neurodevelopmental impairment is defined as a PARCA-R or Bayley cognitive or motor score <70, severe neurological dysfunction (any severe motor impairment, including cerebral palsy of at least grade 2 according to the Gross Motor Function Classification System),^41^ or severe visual or hearing impairment, as previously defined.^18^

Parental psychological assessments are repeated at 6 weeks and 24 months postpartum (figure 2, table 3).

**Table 3:** Primary and secondary study parameters

Primary study parameters	
Twin level	GA at sFGR diagnosis.^5^ EFW discordance at diagnosis.^1 43^ UA Doppler abnormalities.^2^ Presence and diameter of AA anastomoses.^22 44^ Oligohydramnios (DVP<2 cm).^45^ Abnormal DV waveform (absent or reversed flow during the a-wave).^46^ Abnormal placental appearance: ultrasound asymmetry between placental shares, with increased thickness and/or heterogeneity of the smaller twin’s placental share.^44^ Major anomalies (EUROCAT criteria).^5 47^ Cord insertion site.^48^

Secondary study parameters	
I. Prenatal	**Fetal ultrasound** Standard ISUOG parameters: UA/MCA/DV PI and waveforms, MCA PSV, EFW (Hadlock 3: HC, AC, FL), amniotic fluid (DVP), bladder and stomach filling, cord insertions.^49^ Additional ultrasound measures as in tables1 2. **Fetal brain MRI** Presence of IVH, PVL, porencephalic cysts, arterial ischaemic infarction, venous infarct (PVHI), intraparenchymal haemorrhage, ventricular dilation, basal ganglia/thalamic injury, cortical injury, cerebellar injury, migration/gyration disorders, or other abnormalities. **Parental well-being** PCL-5^50^, MAAS^51^, PAAS.^52^ **Maternal** Age, pre-pregnancy BMI, smoking status, obstetric history, comorbidities, medication use, recreational drug or alcohol use, educational level (ISCED). **Gestational/fetal baseline** Gravidity, parity, mode of conception, comorbidity during pregnancy, prenatal screening/diagnostics, fetal sex and non-fatal anomalies.
II. Postnatal	**Labour/delivery** Onset of labour, GA at birth, indication for birth, antenatal steroids, magnesium sulphate, mode of delivery, maternal adverse outcomes. **Placenta** Macroscopic and microscopic assessment (Amsterdam Consensus Criteria),^39^ with additional immunohistochemistry if indicated. **Neonates** APGAR at 1, 5, 10 min. Cord blood arterial/venous pH. Birth weight, HC, anthropometrics. Severe neonatal morbidity.^9^ Neonatal mortality (≤28 days). Cranial US: coronal/sagittal planes (anterior/mastoid fontanelles); CC length, cerebellum, pons, BPD; abnormalities and maturation. Brain volume discordance=(ICV large twin–ICV small twin)/ICV large twin×100. **Parental well-being** PCL-5,^50^ MPAS,^53^ PPAS.^53^
III. Follow-up	**Infant neurodevelopment** PARCA-R and/or Bayley-III/IV.^54 55^ **Parental well-being** PCL-5.^50^

AA, artery-to-artery; AC, abdominal circumference; APGAR, Appearance, Pulse, Grimace, Activity, Respiration; BMI, body mass index; BPD, biparietal diameter; DV, ductus venosus; DVP, deepest vertical pocket; EFW, estimated fetal weight; EUROCAT, European Surveillance of Congenital Anomalies; FL, femur length; GA, gestational age; HC, head circumference; ICV, intracranial volume; ISCED, International Standard Classification of Education; ISUOG, International Society of Ultrasound in Obstetrics and Gynecology; IVH, intraventricular hemorrhage; MAAS, Maternal Antenatal Attachment Scale; MCA, middle cerebral artery; MPAS, Maternal Postnatal Attachment Scale; PAAS, Paternal Antenatal Attachment Scale; PARCA-R, Parent Report of Children’s Abilities-Revised; PCL-5, PTSD Checklist for DSM-5; PI, pulsatility index; PSV, peak systolic velocity; PVL, periventricular leucomalacia; sFGR, selective fetal growth restriction; UA, umbilical artery; US, ultrasound.

### Study outcomes

#### Primary outcome

A composite adverse outcome at the pregnancy level:

- Fetal demise of one or both twins (including subsequent demise after a fetal intervention), and/or
- Induced delivery <32 weeks of gestational age due to perceived fetal distress.

Perceived fetal distress is assessed according to local clinical protocols, as summarised in a recently published multicentre study on outcomes of severe sFGR.^9^ Across centres, fetal indications for earlier delivery commonly include one or more of the following: non-reassuring cardiotocography, significant growth plateau or arrest on the EFW trajectory, worsening Doppler abnormalities such as a reversed a-wave in the ductus venosus, and oligohydramnios. An early onset of sFGR may also contribute to clinical concern.

The candidate predictors (as defined at the time of protocol development) are listed in table 3. The final predictor set will be refined according to the latest literature at the time of analysis.

#### Secondary outcomes

- Perinatal morbidity and mortality.
- Cardiac adaptation in both twins.
- Fetal brain development and neurodevelopment at 24 months.
- Correlation between antenatal and postnatal brain imaging.
- Placental architecture (gross and histological).
- Impact on parental mental health and attachment.
- Impact of management protocol differences across centres on primary outcome.

The secondary study parameters are outlined in table 3.

### Sample size calculation

Sample size was determined using the four-step approach proposed by Riley *et al*.^42^ Based on the most recent published incidences of intrauterine demise and fetal deterioration in sFGR, we estimated a 23% incidence of the primary composite outcome.^1^ Allowing for five key predictors identified a priori, a minimum of 274 twin pairs with complete data is required to ensure model stability and accurate risk estimation. The full sample size calculation can be found in online supplemental materials.

### Data analysis plan

The primary analysis will develop a multivariable logistic regression model predicting the composite outcome at diagnosis. Model performance will be evaluated by discrimination (C-statistic) and calibration (scaled Brier score), with internal validation by bootstrapping.

Secondary analyses will include:

- Longitudinal modelling of ultrasound trajectories using mixed-effects models (continuous variables) and generalised estimating equations (GEE) (categorical variables), with stratification by gestational age at diagnosis (according to the most recent literature-based cut-offs) and umbilical artery Doppler flow type.
- Associations between antenatal imaging and placental pathology.
- Comparison of neurodevelopmental outcomes between co-twins using GEE or paired t-tests.
- Parental well-being was analysed across timepoints using mixed-effects models.
- Comparison of outcomes between outpatient- and inpatient-managed pregnancies.

All tests will be two-sided, with p<0.05 considered significant. Analyses will be conducted in SPSS V.25 (IBM, Chicago, USA) and/or R.

Missing data will be minimised through prospective monitoring, regular quality audits and data queries to participating centres. For the primary composite outcome, missing delivery or fetal outcome data will be actively retrieved where possible; pregnancies with unascertainable primary outcomes will be excluded from the primary prediction model analysis. For missing predictor variables, recovery from source imaging or clinical records will be attempted. If data remain missing, appropriate statistical methods will be applied based on the extent and pattern of missingness, in line with best practice for prediction modelling. Missing questionnaire data will be addressed through reminders; incomplete questionnaires will be excluded from the relevant secondary analyses, with the degree of missingness reported.

### Trial status

Study preparation began in 2022. Recruitment started in March 2023 and was expanded to all centres in June 2024. By December 2025, 209 twin pairs had been enrolled (76% of the target), with approximately 10 new pairs recruited per month. Recruitment will continue until the end of 2026.

### Patient and public involvement

Patients and members of the public were not involved in the scientific design, methodology or conduct of the CONTRAST study. However, a mother who previously gave birth to twins affected by sFGR contributed to the development and refinement of the patient counselling summary flyer used during recruitment. In addition, representatives from the TAPS Support Foundation provided feedback on the wording of participant questionnaires and on communication materials to ensure clarity and accessibility. They also interviewed members of the study team and disseminated information about the study in lay terms on their public website. On completion of the study, a plain-language summary of the results will be made available on the clinical trial registration website and shared through our institutional communication channels for participants and the wider public.

### Ethics and dissemination

The CONTRAST study protocol and informed consent procedures have been approved by the by the institutional review boards or ethical review authority of all participating centres: Leiden University Medical Center (NL81805.058.22), University Hospitals Leuven (S67514), Karolinska University Hospital (2023-04704-01), BCNatal (HCB/2023/0123), Mount Sinai (23-0161-E) and Boston Children’s Hospital (IRB-P00046170). Written informed consent is obtained from all participants prior to inclusion.

Data are managed according to Good Clinical Practice and the General Data Protection Regulation. All information is coded and stored in a secure, web-based electronic case report form (CASTOR). Identifiable information is stored separately and accessible only to local principal investigators. Access to coded data is restricted to study investigators and authorised monitors or supervisory authorities. Data will be stored for at least 15 years after study completion.

Ultrasound images and cardiac clips are pseudonymised and uploaded to CASTOR, with central analysis performed at Leiden University Medical Center. Only coded data are used in analyses, reports and publications.

Findings from this study will be disseminated through presentations at national and international scientific conferences and publications in peer-reviewed journals, in line with the CCMO statement on publication policy. A plain-language summary of the study results will also be published on the clinical trial registration website for participants and the general public. Where appropriate, data may be made available for secondary analyses to address the study objectives, subject to ethical and legal requirements.

## Supplementary material

10.1136/bmjopen-2025-114000 online supplemental file 1
