# Supplementary material for: Predicting outcomes in selective fetal growth restriction of monoChOrioNic Twins: an inteRnAtional observational cohort STudy protocol (CONTRAST study)
Source: BMJ Open. 2026 Feb 24;16(2):e114000. doi: 10.1136/bmjopen-2025-114000 (PMC12933764; doi:10.1136/bmjopen-2025-114000)
Supplement: online supplemental file 1 [file bmjopen-16-2-s001.docx]

**Supplementary materials – sample size calculation**

No previous study has directly reported the incidence of our primary composite outcome. To approximate this, we drew on data from the meta-analysis by Buca et al. (1), which reported incidences of intrauterine fetal demise (IUFD) and fetal deterioration. For the purpose of this calculation, we assumed that 50% of fetal deteriorations would result in iatrogenic preterm delivery. These estimates represented the most comprehensive data available at the time of protocol development.

Estimated incidence of IUFD

| **Gratacós type** | **Type incidence (%)** | **IUFD incidence (%)** |
| --- | --- | --- |
| Type I | 39 | 3.1 |
| Type II | 38.2 | 11 |
| Type III | 22.8 | 9.6 |
| Weighted IUFD incidence | | 7.6 (0.076) |

Estimated incidence of fetal deterioration

| **Gratacós type** | **Type incidence (%)** | **Deterioration (%)** |
| --- | --- | --- |
| Type I | 39 | 16 |
| Type II | 38.2 | 59 |
| Type III | 22.8 | 10 |
| Weighted deterioration incidence | | 31 (0.31) |

| Estimated incidence of the composite outcome:  0.076 + (0.31*0.5) = 0.23 (23%) (assuming 50% of deteriorations lead to iatrogenic preterm delivery) |
| --- |

Sample size calculation according to 4-step approach

Based on five candidate predictors and an estimated composite outcome incidence of 0.23, the required sample size was calculated using the four-step method proposed by Riley et al. (48):

1. Precision of outcome risk (margin of error ≤ 0.05):

$$n= \left( \frac{1.96}{0.05} \right)^{2} 0.23 \left( 1-0.23 \right)=273$$

2. Prediction error (mean absolute percentage error; MAPE of ≤ 0.05), using van Smeden formula (52):

$$n=expexp \left( \frac{-0.508+0.259lnln \left( \emptyset\right)+0.504lnln \left( P \right)-lnln (MAPE)}{0.544} \right)=214$$

3. Shrinkage (S) ≤10%, such that S: ≥ 0.9, Max (R^2^_CS_): 0.66 (see Step 4), anticipated (R^2^_CS_): 0.15:

$$n= \frac{5}{\left( 0.9-1 \right)lnln \left( 1- \frac{0.15}{0.9} \right)}=274$$

4. Optimism in model fit, anticipated R^2^_CS_: 0.099, maximum R^2^_CS_: 0.66, S = 0.75. Assuming n = 100 (arbitrary), events (E) = 23:

$$1) lnL_{null}=23lnln \frac{23}{100} + \left( 100-23 \right)lnln \left( 1- \frac{23}{100} \right)= -54,08$$

$$3) S= \frac{0.099}{0.099+ \partial* 0.066}=0.75$$

$$4) n= \frac{5}{\left( 0.75-1 \right)lnln \left( 1- \frac{0.099}{0.75} \right)}=141$$

Taking the largest estimate from these steps, the study aims to include 274 twin pairs.
