# Supplementary material for: Predicting outcomes in selective fetal growth restriction of monoChOrioNic Twins: an inteRnAtional observational cohort STudy protocol (CONTRAST study)
Source: BMJ Open. 2026 Feb 24;16(2):e114000. doi: 10.1136/bmjopen-2025-114000 (PMC12933764; doi:10.1136/bmjopen-2025-114000)
Supplement: Supplementary file 2 [file bmjopen-2025-114000.reviewer_comments.pdf]

# PEER REVIEW HISTORY

BMJ Open publishes all reviews undertaken for accepted manuscripts. Reviewers are asked to complete a checklist review form and are provided with free text boxes to elaborate on their assessment. These free text comments are reproduced below.

**ARTICLE DETAILS**

**Title (Provisional)**

Predicting outcomes in selective fetal growth restriction of monoChOrioNic Twins: an inteRnAtional observational cohort STudy protocol (CONTRAST-study)

**Authors**

Noll, Anne; Javinani, Ali; Slaghekke, Femke; Haak, Monique C; van Klink, Jeanine; Van der Meeren, Lotte; Lopriore, Enrico; Russo, Francesca; Aertsen, Michael; Shamshirsaz, Alireza; Shinar, S.; Bennasar, Mar; Tiblad, Eleonor; Herling, Lotta; Lewi, Liesbeth; Verweij, E.J.T Joanne; Study group, CONTRAST

---

**VERSION 1 - REVIEW**

---

| | |
|---|---|
| **Reviewer** | **1** |
| **Name** | **Soni, Shelly** |
| **Affiliation** | **The Children's Hospital of Philadelphia, Fetal Diagnosis and Therapy** |
| **Date** | **29-Nov-2025** |
| **COI** | **None** |

---

"Predicting outcomes in selective fetal growth restriction of monochorionic twins: an international observational cohort study (CONTRAST-study)"

Thank you for this opportunity to review this manuscript that is a prospective, multicenter international cohort protocol aimed at evaluating a prediction model for adverse outcomes in monochorionic diamniotic twin pregnancies complicated by selective fetal growth restriction (sFGR). The topic is clinically important, timely, and highly relevant. The authors report a very comprehensive and well-designed protocol. Strengths include compelling clinical rationale, extensive and clearly articulated study design, and the prospective international collaboration. The protocol has some limitations that require clarification –

- How do authors define "perceived fetal distress"

- What was the rationale for selecting 28 weeks' gestational age as the inclusion cutoff? I would anticipate a wider range of outcomes if such a late gestational age is selected that may confuse the results.

- Why was a discordance threshold of ≥20% chosen as an inclusion criterion? This is not a commonly used definition in the literature. Please cite a reference.

o Please explain if the predictive model will incorporate UA Dopplers as well. If so, please specify in the paper

- How do the authors define a distended bladder?

- Table 4: Please specify a precise definition of abnormal DV waveform and placental dichotomy

Some other minor comments

Please specify how missing data will be handled.

Will different centers use the same kind of ultrasound and MRI machines.

Who will be reading and assessing cardiac clips and neuro MRI.

---

## VERSION 1 - AUTHOR RESPONSE

**Reviewer's Comments:**

**Thank you for this opportunity to review this manuscript that is a prospective, multicenter international cohort protocol aimed at evaluating a prediction model for adverse outcomes in monochorionic diamniotic twin pregnancies complicated by selective fetal growth restriction (sFGR).**

**The topic is clinically important, timely, and highly relevant. The authors report a very comprehensive and well-designed protocol. Strengths include compelling clinical rationale, extensive and clearly articulated study design, and the prospective international collaboration. The protocol has some limitations that require clarification.**

We are grateful to the reviewer for their detailed evaluation of our study protocol, their encouraging comments, and the constructive feedback. We appreciate the recognition of the clinical importance of this work and the strengths highlighted regarding the study design and international collaboration. We address each of the reviewer's comments in detail below.

**- How do authors define "perceived fetal distress"**

a. The reviewer raises an important point. Because this is a prospective observational multicenter cohort study, full harmonization of all clinical management protocols across participating centers was not feasible. While imaging and data collection procedures are standardized, centers retain their local criteria for timing of delivery. As such, "perceived fetal distress" reflects clinical judgement within established center-specific protocols. However, across centers there is substantial expert consensus regarding the fetal indications that typically prompt early delivery in sFGR, as we recently reported in the ALIGN study (Supplementary Materials).

b. We clarified this in the manuscript by adding a footnote to the definition of the primary composite outcome. The footnote now references the ALIGN study and summarises the fetal indicators commonly considered signs of fetal compromise across centres. This clarifies the concept without implying artificial uniformity in management practices.

- Induced delivery <32 weeks of gestational age due to perceived fetal distress*.

"* Perceived fetal distress is assessed according to local clinical protocols, as summarized in a recently published multicenter study on outcomes of severe sFGR (1) Across centers, fetal indications for earlier delivery commonly include one or more of the following: non-reassuring cardiotocography (CTG), significant growth plateau or arrest on the EFW trajectory, worsening Doppler abnormalities such as a reversed a-wave in the ductus venosus (DV), and oligohydramnios. An early-onset of sFGR may also contribute to clinical concern."

c. Study outcomes, primary outcome section, page 9.

**- What was the rationale for selecting 28 weeks' gestational age as the inclusion cutoff? I would anticipate a wider range of outcomes if such a late gestational age is selected that may confuse the results.**

a. This point provides an opportunity to further explain the reasoning behind the selected cutoff.

   Earlier gestational age at sFGR diagnosis is a well-established risk factor for adverse perinatal outcome (2, 3). However, there is no universally accepted gestational age threshold that distinguishes "early" from "late" sFGR, and cut-offs used in the literature vary are arbitrary chosen. Restricting inclusion to (very) early gestational ages would enrich the cohort for severe disease and poor outcomes, but would limit applicability of the prediction model to the milder and more prevalent forms of sFGR encountered in clinical practice.

   We therefore chose an upper inclusion limit of 28 weeks' gestation to capture a broader and clinically relevant spectrum of sFGR severity. This approach also accounts for real-world referral patterns, where delays in referral may lead to diagnosis later in gestation despite earlier disease onset. Importantly, heterogeneity in disease severity is not considered a limitation for prediction modelling but rather a prerequisite for developing a model that is generalizable across the full clinical spectrum of sFGR.

   We do not expect inclusion up to 28 weeks to confuse the results, as gestational age at diagnosis is explicitly included as a candidate predictor in the model. This allows differences between earlier- and later-onset sFGR to be accounted for analytically. Where appropriate, gestational age–based stratification or sensitivity analyses (e.g. earlier versus later diagnosis) will be performed.

a. The following sentence was added to clarify the inclusion criteria:

   "The upper inclusion limit of 28 weeks' gestation was selected to capture both early and later presentations of sFGR, thereby enhancing the clinical relevance and generalizability of the prediction model."

b. Methods, Eligibility, page 4

**- Why was a discordance threshold of ≥20% chosen as an inclusion criterion? This is not a commonly used definition in the literature. Please cite a reference.**

a. The reviewer raises an important point, as the definition of sFGR remains an area of active debate. We acknowledge that multiple definitions are currently used in the literature, including those combining EFW discordance with centile-based criteria (4-6).

   However, the added value of more complex, centile-based definitions has been questioned. For example, Brock et al. demonstrated that, although the Delphi consensus criteria increase the number of pregnancies classified as sFGR, they do not improve identification of pregnancies at increased risk of composite perinatal morbidity and mortality compared with simpler definitions based on EFW discordance and fetal size alone (7). In addition, centile-based definitions also introduce more room for variability, as fetal growth centiles depend on the growth chart applied (singleton versus twin, population-based versus customized) and how first-trimester dating is performed (e.g. smaller, larger, or average crown–rump length).

   Using EFW discordance as the primary inclusion criterion therefore has the advantage of being independent of the growth chart applied, facilitating consistent inclusion across multiple international centers. A threshold of ≥20% EFW discordance has been shown to be associated with increased perinatal risk, while remaining pragmatic and applicable in daily clinical practice (8-10).

   A recent article from Giorgione et al. demonstrated that intertwin EFW discordance is the most reliable predictor of adverse perinatal outcome, outperforming both singleton and twin-specific centile-based growth charts for monochorionic twins. The authors state that an EFW discordance of 15% yields the highest predictive performance at a 20% false positive rate (FPR) cut-off. A discordance of 20% (21% in their supplementary table) yields a FPR cut-off of 10%, and also a very good predictive performance. The composite outcome that the authors use differs from ours however, in terms of gestational age of preterm birth (ours is more strict) (11).

b. We have clarified the rationale for using an EFW discordance threshold of ≥20% in the *Eligibility* section and added supporting references.

   "This sFGR definition enables consistent inclusion across centers regardless of growth chart usage and aligns with clinical practice, whereas the more complex Delphi consensus criteria (6) have shown limited adoption and similar predictive accuracy (7). Recent data further support intertwin EFW discordance as a stronger predictor of adverse outcome than centile-based definitions in monochorionic twins (11). An EFW discordance threshold of ≥20% has been shown to be associated with increased perinatal risk and robust predictive performance, allowing consistent application across centers. The upper inclusion limit of 28 weeks' gestation was selected to capture both early and later presentations of sFGR, thereby enhancing the clinical relevance and generalizability of the prediction model."

c. Methods, Eligibility, page 4

**- Please explain if the predictive model will incorporate UA Dopplers as well. If so, please specify in the paper**

a. UA Doppler Dopplers are indeed well-established prognostic markers in sFGR (1, 12-14), and are therefore important candidate predictors for adverse outcomes.

   We confirm that UA Doppler abnormalities are included among the candidate predictors evaluated for the predictive model. As outlined in Table 4, UA Doppler abnormalities are listed

as twin-level candidate predictors for the primary composite outcome. In accordance with best practice for prediction modelling, the final predictor set will be determined at the time of model development, informed by data availability, statistical performance, and contemporary literature; however, UA Doppler parameters are expected to remain key candidate predictors.

b. We have moved the section describing candidate predictors to directly follow the 'Primary outcome' section to improve clarity and logical flow.

c. Methods, Study parameters, Table 4 (Primary study parameters (twin level), page 10

**- How do the authors define a distended bladder?**

a. Clarification of this definition is appropriate and has now been provided. In line with the referenced diagnostic criteria for TTTS, a "distended bladder" is defined qualitatively rather than by a specific size or volume. It is described as a bladder that is persistently visible and subjectively enlarged on ultrasound, reflecting polyuria and hypervolemia in the recipient twin. No universally accepted quantitative threshold is provided in the literature (15, 16).

b. We have clarified this definition in as follows:

"TTTS is defined by the presence of discordant amniotic fluid volumes, with a maximum vertical pocket (MVP) >6 cm before 16 weeks, >8 cm at 16–20 weeks, or >10 cm after 20 weeks in one sac, and <2 cm in the co-twin, accompanied by a distended bladder (defined as persistently visible and subjectively enlarged on ultrasound) in the recipient and an absent or small bladder in the donor twin."

c. Methods, Eligibility, page 4

**- Table 4: Please specify a precise definition of abnormal DV waveform and placental dichotomy**

a. We agree with the reviewer that both parameters require clearer definitions to ensure consistent interpretation.

An abnormal DV waveform is well defined in the literature as absent or reversed flow during the a-wave (atrial contraction) and is associated with fetal cardiac compromise and adverse perinatal outcome (1, 17) .

The term *placental dichotomy* may be interpreted inconsistently and may overlap conceptually with color placental dichotomy as used in the context of TAPS. In this study, the intended parameter rather reflects ultrasound evidence of asymmetry in placental morphology (gross appearance, thickness) between the two placental shares, rather than a color-based phenomenon (18).

b. We have clarified and revised the terminology in Table 4 as follows:

- Abnormal DV waveform (absent or reversed flow during the a-wave) (17)

In addition, we have replaced the term *placental dichotomy* with "(abnormal) placental appearance (smaller twin)" throughout the protocol, and defined it as follows:

- Abnormal placental appearance: ultrasound asymmetry between placental shares, with increased thickness and/or heterogeneity of the smaller twin's placental share.

c. Methods, Table 4, page 10

Some other minor comments

**- Please specify how missing data will be handled.**

a. This methodological aspect has now been described in greater detail. Given the multicenter prospective design, we will minimize missingness through standardized data collection and central monitoring, and targeted queries to participating sites. Nonetheless, missing data may occur (incomplete predictor measurements, incomplete follow-up, or unavailable imaging at some sites, missing questionnaires).

   We will minimize missing data through prospective monitoring, regular quality audits, and data queries to participating centers.

   For the primary composite outcome, sites will be contacted to retrieve missing delivery and fetal outcome data wherever possible. Participants for whom the primary outcome cannot be ascertained will be excluded from the primary prediction model analysis.

   For missing predictor data, we will first assess whether values can be recovered from source imaging or clinical records. If predictor data remain missing, appropriate statistical methods for handling missing data will be applied in accordance with best practice for prediction modelling and the extent and pattern of missingness. Where applicable, imputation-based approaches will be considered, and sensitivity analyses may be performed to evaluate the impact of missing data on model performance.

   For missing questionnaire data, participants and/or partners will be reminded to complete the questionnaires. If questionnaires remain incomplete, these data will be excluded from the relevant secondary analyses, and the extent of missingness will be reported.

b. The following has been added to the manuscript:

   "Missing data will be minimized through prospective monitoring, regular quality audits, and data queries to participating centers. For the primary composite outcome, missing delivery or fetal outcome data will be actively retrieved where possible; pregnancies with unascertainable primary outcomes will be excluded from the primary prediction model analysis. For missing predictor variables, recovery from source imaging or clinical records will be attempted. If data remain missing, appropriate statistical methods will be applied based on the extent and pattern of missingness, in line with best practice for prediction modelling. Missing questionnaire data will be addressed through reminders; incomplete questionnaires will be excluded from the relevant secondary analyses, with the degree of missingness reported."

c. Data analysis plan, page 12.

**- Will different centers use the same kind of ultrasound and MRI machines.**

a. This is an important methodological consideration, as variability in imaging equipment could theoretically affect image acquisition and measurement. Given the international, multicenter nature of the study, complete uniformity in imaging platforms is not feasible.

b. We clarify that the majority of centers use the same ultrasound platform, and that imaging acquisition is standardized through harmonized protocols and quality audits. Differences in MRI field strength are documented and considered during analysis.

   Specifically, five of the six participating centres use Voluson E22 ultrasound machines (GE Healthcare), while one centre uses Canon APLIO i800/i700 systems (Canon Medical Systems). Fetal MRI scanners vary by center (e.g. 1.5T or 3T), and scanner characteristics are recorded for each examination. We do not expect these differences to materially influence study outcomes, as image acquisition follows standardized protocols and measurements are performed locally by experienced operators, with central review and quality assurance.

   We have added the following text to the footnote of Table 2:
   "Five centers use Voluson E22 machines (GE Healthcare) for ultrasound acquisition and one center uses Canon APLIO i800/i700 machines (Canon Medical Systems). Fetal MRI scanner characteristics are recorded per examination. Standardized acquisition protocols and regular quality audits are used to ensure consistency across centers."

c. Methods, Table 2 (footnote), page 9

**- Who will be reading and assessing cardiac clips and neuro MRI.**

a. We agree that clarity on image assessment is important for transparency. While image acquisition is performed locally, cardiac clips evaluation is carried out by our experienced subspecialist (LH), in collaboration with professor MH, to ensure consistency and quality. Fetal MRI is performed locally, but monitored by our experienced pediatric neuroradiologist (EM).

**Other changes:**

- A few textual changes are adapted for clarity and conciseness:
  - This sFGR definition ~~simplified definition~~ enables (Methods, Eligibility, page 4)
  - ~~187~~205 twin pairs (latest inclusion number) (Trial status, page 12)

**References:**


1. Noll ATR, van Hoogstraten A, Nulens K, van Geloven N, Van Mieghem T, Shinar S, et al. Outcome of monochorionic diamniotic twin pregnancy with selective fetal growth restriction and continuous or intermittent absent or reversed end-diastolic umbilical artery flow: international multicenter cohort study. Ultrasound Obstet Gynecol. 2025;66(1):41–50.
2. Curado J, Sileo F, Bhide A, Thilaganathan B, Khalil A. Early- and late-onset selective fetal growth restriction in monochorionic diamniotic twin pregnancy: natural history and diagnostic criteria. Ultrasound Obstet Gynecol. 2020;55(5):661–6.
3. Couck I, Ponnet S, Deprest J, Devlieger R, De Catte L, Lewi L. Outcome of monochorionic twin pregnancy with selective fetal growth restriction at 16, 20 or 30 weeks according to new Delphi consensus definition. Ultrasound Obstet Gynecol. 2020;56(6):821–30.
4. Khalil A, Rodgers M, Baschat A, Bhide A, Gratacos E, Hecher K, et al. ISUOG Practice Guidelines: role of ultrasound in twin pregnancy. Ultrasound Obstet Gynecol. 2016;47(2):247–63.

5. National Institute for Health and Care Excellence: Guidelines. Twin and Triplet Pregnancy. London: National Institute for Health and Care Excellence (NICE)

Copyright © NICE 2019.; 2019.
6. Khalil A, Beune I, Hecher K, Wynia K, Ganzevoort W, Reed K, et al. Consensus definition and essential reporting parameters of selective fetal growth restriction in twin pregnancy: a Delphi procedure. Ultrasound Obstet Gynecol. 2019;53(1):47–54.
7. Brock CO, Bergh EP, Johnson A, Ruano R, Andrade EH, Papanna R. The Delphi definition for selective fetal growth restriction may not improve detection of pathologic growth discordance in monochorionic twins. Am J Obstet Gynecol MFM. 2022:100561.
8. Breathnach FM, McAuliffe FM, Geary M, Daly S, Higgins JR, Dornan J, et al. Definition of intertwin birth weight discordance. Obstet Gynecol. 2011;118(1):94–103.
9. Talbot GT, Goldstein RF, Nesbitt T, Johnson JL, Kay HH. Is size discordancy an indication for delivery of preterm twins? Am J Obstet Gynecol. 1997;177(5):1050–4.
10. Multifetal Gestations: Twin, Triplet, and Higher-Order Multifetal Pregnancies: ACOG Practice Bulletin, Number 231. Obstet Gynecol. 2021;137(6):e145–e62.
11. Giorgione V, Lopian M, Trapani M, Brutto M, Ferrante MG, Bhide A, et al. Adverse perinatal outcomes in twins: comparison of intertwin fetal size discordance vs singleton and twin fetal growth charts. Ultrasound Obstet Gynecol. 2025.
12. Gratacós E, Lewi L, Muñoz B, Acosta-Rojas R, Hernandez-Andrade E, Martinez JM, et al. A classification system for selective intrauterine growth restriction in monochorionic pregnancies according to umbilical artery Doppler flow in the smaller twin. Ultrasound Obstet Gynecol. 2007;30(1):28–34.
13. Buca D, Pagani G, Rizzo G, Familiari A, Flacco ME, Manzoli L, et al. Outcome of monochorionic twin pregnancy with selective intrauterine growth restriction according to umbilical artery Doppler flow pattern of smaller twin: systematic review and meta-analysis. Ultrasound Obstet Gynecol. 2017;50(5):559–68.
14. Shinar S, Xing W, Pruthi V, Jianping C, Slaghekke F, Groene S, et al. Outcome of monochorionic twin pregnancy complicated by Type-III selective intrauterine growth restriction. Ultrasound Obstet Gynecol. 2021;57(1):126–33.
15. Quintero RA, Morales WJ, Allen MH, Bornick PW, Johnson PK, Kruger M. Staging of twin-twin transfusion syndrome. J Perinatol. 1999;19(8 Pt 1):550–5.
16. Khalil A. Modified diagnostic criteria for twin-to-twin transfusion syndrome prior to 18 weeks' gestation: time to change? Ultrasound Obstet Gynecol. 2017;49(6):804–5.
17. Monaghan C, Kalafat E, Binder J, Thilaganathan B, Khalil A. Prediction of adverse pregnancy outcome in monochorionic diamniotic twin pregnancy complicated by selective fetal growth restriction. Ultrasound Obstet Gynecol. 2019;53(2):200–7.
18. Couck I, Deprest J, Lewi L. How to better distinguish between Type II and III selective fetal growth restriction in monochorionic twin pregnancies? Fetal Diagn Ther. 2022.

---

## VERSION 2 - REVIEW

---

| | |
|---|---|
| **Reviewer** | **1** |
| **Name** | **Soni, Shelly** |
| **Affiliation** | **The Children's Hospital of Philadelphia, Fetal Diagnosis and Therapy** |
| **Date** | **20-Jan-2026** |

## COI

---

Just some minor comments –

Abstract:

The authors refer to “monochorionic twins.” For clarity and consistency with the rest of the manuscript, this should be specified as monochorionic diamniotic twins, with MCDA introduced as an abbreviation at first mention

Maternal and ?parental - do the authors mean paternal

Introduction:

“As no curative treatment exists, timely delivery remains the only intervention, making accurate outcome prediction essential” – this is an incomplete statement as there is salvage therapy available (e.g., selective reduction or cord occlusion) which is sometimes employed to improve survival of one twin at the expense of the other. The authors should acknowledge this to more accurately reflect current clinical practice.

The rationale for inclusion up to 28 weeks’ gestation is reasonable. However, readers may benefit from - Stratification of outcomes by gestational age at diagnosis and a clear definition of “early” and “late” onset sFGR.

---

## VERSION 2 - AUTHOR RESPONSE

**Reviewer's Comments:**

**Abstract:**
**The authors refer to “monochorionic twins.” For clarity and consistency with the rest of the manuscript, this should be specified as monochorionic diamniotic twins, with MCDA introduced as an abbreviation at first mention**

a) We agree with the reviewer that consistent terminology should be used throughout the manuscript.

b) We have revised the abstract to specify “monochorionic diamniotic twins” and introduced the abbreviation MCDA at first mention.

c) Abstract, page 2.

**Maternal and ?parental - do the authors mean paternal**

We understand the reviewer’s interpretation. We intentionally use the gender-neutral term “parental” rather than “paternal” to inclusively refer to all non-gestational parents, including prospective parents of the same sex.

**Introduction:**
***“As no curative treatment exists, timely delivery remains the only intervention, making accurate outcome prediction essential”* – this is an incomplete statement as there is salvage therapy**

**available (e.g., selective reduction or cord occlusion) which is sometimes employed to improve survival of one twin at the expense of the other. The authors should acknowledge this to more accurately reflect current clinical practice.**

a) We agree with the reviewer that fetal interventional options may be considered in selected severe cases. As these interventions aim to preserve the survival of one twin rather than to treat sFGR affecting both fetuses, we did not previously categorize them as curative treatment.

b) We have now explicitly acknowledged these interventional options in the Introduction by revising the text as follows:

"Although fetal interventions such as selective reduction or cord occlusion may be considered in selected severe cases, no curative treatment exists. Timely delivery therefore remains the principal intervention, making accurate outcome prediction essential."

c) Introduction, page 3.

**The rationale for inclusion up to 28 weeks' gestation is reasonable. However, readers may benefit from - Stratification of outcomes by gestational age at diagnosis and a clear definition of "early" and "late" onset sFGR.**

a. We appreciate the reviewer's suggestion to explicitly address stratification by gestational age at diagnosis and to clarify the concept of early- versus late-onset sFGR.

b. We have amended the Data analysis plan to specify that secondary analyses will include stratification by gestational age at diagnosis, in addition to other clinically relevant parameters such as umbilical artery Doppler flow type.

We deliberately did not prespecify a fixed gestational age cut-off for "early" versus "late" sFGR, as definitions used in the literature (e.g. before vs after 24 weeks) are largely consensus-based and lack a clear biological demarcation (1, 2). To ensure methodological rigor and contemporaneity, the final stratification will be informed by the most recent evidence and consensus available at the time of analysis. If no new insights emerge, we will adhere to the most widely accepted consensus-based definitions.

Secondary analyses will include:

- Longitudinal modeling of ultrasound trajectories using mixed-effects models (continuous variables) and generalized estimating equations (GEE) (categorical variables), with stratification by GA at diagnosis (according to the most recent literature-based cut-offs) and UA Doppler flow type.

c. Data analysis plan, page 11.

**Other changes:**

- A few textual changes are adapted for clarity and conciseness:
  - ~~187~~209 twin pairs (latest inclusion number) (Trial status, page 12)

**References:**

1. Curado J, Sileo F, Bhide A, Thilaganathan B, Khalil A. Early- and late-onset selective fetal growth restriction in monochorionic diamniotic twin pregnancy: natural history and diagnostic criteria. Ultrasound Obstet Gynecol. 2020;55(5):661–6.
2. Couck I, Ponnet S, Deprest J, Devlieger R, De Catte L, Lewi L. Outcome of monochorionic twin pregnancy with selective fetal growth restriction at 16, 20 or 30 weeks according to new Delphi consensus definition. Ultrasound Obstet Gynecol. 2020;56(6):821–30.
